# Woman in Her Social Relations, past and Present

**Published:** 1856-10-01

**Authors:** 


### Art. II.—WOMAN IN HER SOCIAL RELATIONS, PAST AND PRESENT.

In an early number of this Journal, woman was considered psychologically; let us now shortly view her in her social and political relations. The student of history, when once his attention is called to the fact, must regard with some surprise the scanty mention made of the female sex in those annals which profess to treat of the birth and growth of the nations of the earth. Where history merges into the golden land of fable, he will indeed find women playing a conspicuous and important part in the affairs of life; it was for a woman that Troy was ten years besieged by all the myrmidons of assembled Greece; in the quarrel caused by a woman that the fields of upper air resounded with the conflict of angry deities, while Scamander flowed purple with the blood of heroes. It was for wrong done to a woman that Tarquin and his race were expelled the Roman throne; it was a woman who rallied the energies of Britain, and vanquished the descendants of Brutus, at what is now our modern town of Colchester, till forced to yield herself in a less victorious field. But lower down the stream of each nation's life, the traces of women so utterly disappear, that the student is tempted to ask himself whether there really were any wives, mothers, and maidens, in those days, bearing the same equal numerical proportion to husbands, fathers, and youths, as they do at the present day; or whether armed men did not rather spring full panoplied from the blood of fallen warriors, to hate, to fight, and to destroy, to make experiments in legislation, and to matriculate in various forms of legal and illegal murder, till they too gave place to the generation which should succeed and do likewise.

In no book is this utter absence of the female element more strangely noticeable than in the most erudite and philosophical production of late years—Mr. Grote's "History of Greece." In twelve thick volumes, containing the result of the labour of a life, full of carefully arranged information concerning the most fruitful epochs of ancient time—the years in which not only politics, but literature and the arts attained a development to which we may well question ourselves if our own be indeed superior, we find no more mention of women than that Sappho had a great gift of song, that Socrates was afflicted by a cross wife, and that Pericles rejoiced in the love of that one woman of old Athenian days, whose majestic and beautiful image stands out all the more prominently from the field of ancient history, because it is so solitary, so entirely unique. Then how many pages of Gibbon or of Hume may the reader devour, asking vainly, "Were there any women; what did they do, what did they say?" This one perhaps, went to rescue her husband from prison, or that one died a martyr, or a third held gallantly her lord's castle against the besiegers, till he could hasten back to the rescue from some distant war. Of the first eight centuries A.D., we chiefly learn that Alfred's mother taught him to read, and that Christianity was welcomed into England by "a Queen of Kent," witness any number of cartoons by ambitious British artists. In France we have Brunhilda, wife to Sigebert (575), and the queen of Charlemagne, that fair Fastrada, for whom he mourned with such inconsolable bitterness; but here we trench once more upon the world of fiction, for the story runs off into magic rings and such veracious ornaments of history. As to Haroun al Raschid, the third in the illustrious trio of monarchs who ennobled the ninth century, his very name is synonymous with all manner of wild and witty fable, and whatever were the accredited exploits of that great hero of eastern romance, even Professor Smyth could not have extracted a single golden grain of truth from the chronicles in which

> "The tide of time flow'd back with me,
> The forward flowing tide of time:
> For it was in the golden prime
> Of good Haroun al Raschid."

Yet through all these centuries before and after Christ, the human race was, as now, wrought up of two inseparable elements: "male and female created He them," and the influence of the woman penetrated subtlely into every cranny of the social edifice. The religious ideas of each age were fostered and diffused by women as surely as they are at the present day;—the morals of the time were such as their education, their consent, allowed in practice;—the universal ideal of the nation could be neither higher nor lower than the level of that all-pervading, all-influential body of female opinion. A moment's thought will show this: no idea could prevail in a society, were it in every household a matter of question and disunion; the women must either oppose or succumb to any given dogma, large or small. If they oppose, they ultimately modify; if they succumb, they enforce it with the weight of prejudice added to that of reason. Whether it be by dint of beauty, of tears, or of *finesse*, the uncultivated woman is equal in fact to the uncultivated man: if he loves her, she sways him; if he beats her, she conspires against him; if he denies her a voice in the council, she takes care to exercise it at home. If he misuse her fine nature, he finds that he has created for himself a torment more intense, because nearer, than any other can be; his wine is turned into vinegar, his roses are changed into scorpion's tongues, so that the silence of history in reference to women, while it is amply significant of the public estimation in which they were held at any given epoch, is in no wise significant of that natural and indestructible balance which the Creator made when He "formed man in His own image, male and female created He them."

We are therefore cast back upon those traditions which we have before alluded to as flowing down to us from the hidden sources of history proper, through many a picturesque and winding way; and these, however little they may represent the outward fact, do in reality contain the very kernel of the past life of a nation, giving at least the ideal to which it was directed, clothed in the costume and surrounded by the accessories of those historical epochs in which the traditions developed. As we ascend into the heroic age, and ask of the legend, the epic, and the drama, what manner of men the primeval epoch bore, we find the two sexes playing nearly equal parts in the game of existence ; a grand heroic game as it is painted ! It is true that wives and daughters were strictly reduced in theory to a condition of obedience—that Iphigenia bows before the knife, while contending warriors wrangle over the fate of Helen, as though she were a stock or a stone ; but we nevertheless find them the centres of great movements, the inspirers of great passions, holding in their slender hands the thread of many a mighty fate.

"Thebes or Pelop's line,
Or the tale of Troy divine—"

So that such legends represent life more truly than any number of chronicles of wars—than any diagrams of political constitutions; for it is not the outer framework of the history of a country which can give food for thought, or teaching for practice ; it is only the inner reality of life as it was lived among its people, which can do this : knowledge of what were the moral elements of their society; how they moved and spoke and dressed, how they wooed and wedded, and buried their dead ; of what manner was their worship, and upon what rested their hope and their faith.

And when, by the help of these poetical pictures, we come to ask ourselves what, in the earliest times of which we can form any image, was the relation of women to society considered in the aggregate *political* sense, we are inclined to say that it possessed a sort of rough equality with that of men; that where political constitutions were so simple that to wield arms under some strong and valiant hero, and to take occasional part in the deliberations of camp or council, were the chief political duties of the husband, the wife who bore and reared the young warriors, and took her full half share in the simple exertions demanded by the needs of primitive existence, could hardly be said to hold an inferior place, or be less of a citizen than he himself.

But when society became more complex, when distinctions of rank were defined and cemented by interchange of service and privilege, when Lynch law no longer prevailed for the redressing of evils, but the Areopagus and the Forum, the Witenagemote and the Parliament, came to supersede the rough and ready machinery of earlier ages,—then, amid the linked spheres of what we consider a more perfect political system, women found no place. The sex cannot be said to have held any relation to the state during the historical eras, except in that singular and anomalous instance when, as in our own country at the present day, a woman filled the highest post in the realm, presiding, in the capacity of sovereign, over that government in whose behalf, had she been of lower degree, she would not even have possessed the privilege of giving a vote towards the election of the meanest official.

Parallel with the rise of elaborate political systems, sprang into being those arts and sciences which became with men the subject of long study, and the material of severe and learned professions. Not only were women (either by nature, law, or custom) debarred from the profound investigation of the one, and the careful practice of the other, but even amateur attainment was considered apart from their sphere of duty, so that a still further division was created between the sexes, and the pair who in barbarian life pursued in divers manners, but in strict conjunction, the simple aims of physical existence, came at length to have scarcely any interests in common, save those which spring directly or indirectly from love. The student finds himself, therefore, reduced to ask how the laws which were framed by men bore upon the other sex, happy if a vivid imagination, the power of seizing upon a few meagre and scattered hints, combined with subtle analysis of the state of feeling which dictates any given law, and into which such a law must again react, afford him any help in reconstructing for himself something like a real colour-picture of that past which lies buried, for the casual reader, under a mountain of dates and names and dry records of facts, bearing indeed on certain interesting problems of legislation, but having little relation to any of the more vital questions affecting the moral growth of humanity. Not that we would under-estimate the value of conclusions in favour of democracy, of equal taxation, and impartial criminal law; yet our most valuable facts on such subjects must be drawn from modern *social* investigation, not from historical records or abstract deduction. It sometimes seems to us that history, as at present availably written (for it requires somewhat of the imagination of the poet to seize and employ, like Carlyle, the more rich and subtle hues of historical truth), and politics as at present conducted, are nearly as abstract as mathematics themselves; that the fate of nations is played with by those in whose hands lie their more external destinies, much after the manner of a game of chess. The soldiers are but as animated pawns, Russia against France becomes a scientific question of weight and resistance; but descend into those home regions from whence the material of the war is drawn, from whence the bare facts of the chronicle are distilled, and how would the scene change; how vivid, how full of life, of the pain of parting and the joy of meeting, it would become. The automata are animated, it is no longer the nation merely, no longer Britannia with her lion, or Gallia with her cock and tricolor. The women start into their pristine equality of interest; they are the mothers of soldiers and sailors, and assemble by hundreds, weeping in the morning twilight to see them depart; they are the wives of the bread-winners, and each tax, each change in the administration, sends a subtle thrill from the Lords and Commons through the needs, the sympathies, and the sorrows of the women to the farthest corner of the land. Let us therefore inquire what have been the laws relating to women in various ages and countries, that being a very rough, but almost our only means of attaining to something like an idea of the real social condition of the sex.

We find in Michaelis's "Laws of Moses," first volume, 34th chapter of the English translation, by Alexander Smith, D.D., various particulars collected together concerning the legal position of Jewish women. Among the Hebrews, wives were commonly bought, according to the practice of the East. The case was the same among the Arabs and Syrians. In the language of the latter, *mechiro*, or the *sold*, is equivalent to the *espoused*, just as in the German Chronicles of the middle ages we find it stated that A B *bought* C D, that is, married her. The Arabs have, along with their religion, carried this practice far into Asia, and established it in countries where, before their conquest, it had no footing; and Arireux, in his Travels, says that among the Mahometans there are three sorts of wives—*married*, *bought*, and *hired*. Polygamy, in raising the demand for women, sets as it were a price upon them; and as in Jewish days a girl was under the control of her father and brothers, this price would naturally be exacted. It does not appear, however, that the rule was invariable, since Sarah and Rebecca seem to have held positions of power and freedom incompatible with being the subjects of sale; and the presents which Abraham's servant brought with him when he came to seek a wife for Isaac, were, however rich, presents, and not purchase-money. But we find Jacob giving seven years' labour as the price of Rachel; and even in the most poetical of the marriages recorded in the Scriptures, that of Ruth, we find the spirit of the contract to prevail externally; for Boaz called the elders together, telling them that he had *purchased* Ruth to be his wife. A large number of women, both Jewish, and of foreign birth taken in war, were virtually slaves, and occupied definite legal positions as servants or as handmaids. These were all in a measure protected by the Mosaic regulations. For instance, a master who did not choose to marry his Israelitish handmaid himself, was not allowed to retain her, deprived of the joys of domestic life, but was obliged when any one—such, for instance, as a near relation or an intending husband—expressed a wish of redeeming her, to let her go at reasonable ransom. The whole sex was, however, in a state of miserable legal slavery: where there were sons, they seem to have exercised a certain authority over the fate of their sisters, even during their father's lifetime; where there were none, "an heiress durst not marry without her tribe, and seldom did marry out of her family." Again, Jephtha, having offered to the Lord as a burnt-offering the first living thing which should meet him on his homeward return from the war, is welcomed by his daughter "with timbrels and with dances. She was his only child: beside her he had neither son nor daughter." But he having opened his mouth to the Lord, could not go back, and appropriated her in fulfilment of the vow, with much lamentation indeed, but no apparent opposition on the part of her companions. It was not her war, nor her vow; but being as much her father's property as a sheep or a goat, she came under the category of "the first living thing."

Egyptian manners were very different. Herodotus affirms that throughout Egypt it was customary to marry only one wife; and "if the authority of Diodorus can be credited, women were indulged with greater privileges in Egypt than in any other country." We learn from Wilkinson various details—such as that women reigned as sovereigns; that they were not secluded as in Greece; part of the worship of the gods was entrusted to their management, and that not in processions and ceremonies, but under enrolment in regular priesthoods; "and if we are not correctly informed of the real extent and nature of their duties, yet, since females of the noblest families, and princesses, as well as the queens themselves, esteemed it an honour to perform them, we may conclude the post was one of the highest to which they could aspire in the service of religion." It is worthy of remark, that direct mention of priestesses is made in that Rosetta stone which is one of the principal antiquarian objects of interest in our British Museum. The pictorial illustrations which we possess of Egyptian life give us more idea of social customs than antiquity generally vouchsafes to the student; and we may mention in passing that we see in the strange frescoes which that clear climate has preserved, various indications of the domestic avocations of the women, of their weaving, and use of the distaff, of their practice of music, and sometimes, unfortunately, of their being too much addicted to convivial entertainments!

In an article contained in the *Westminster Review* for October, 1855, the reader will find a variety of curious information concerning the women of Arabia, of China, the Indian Archipelago, and many other "far contrees" of this habitable globe, where men and women are living together under various laws, all characterized by the same general feature, that of regarding the latter as articles of property. The Pagan Arabs not only bought their wives to the number of eight or ten, "but actually exchanged them with each other." Mahomet, reducing the number, did away altogether with the right of exchange; but "the father still disposes of the daughter in marriage, and a payment to the father or guardian is necessary to legalise the marriage, and the least sum allowed by the law is ten dirhems, or drams, of silver—about five shillings." In other respects, the exclusive habits of Mohammedans in regard to women are well known. The Hindu laws also make marriage a matter of bargain; and that from the earliest times of which any record is obtainable. Mr. Mill, the historian of India, "is convinced that the life of Hindu women is a life of the most abject degradation." This conclusion is based on the study of the Hindu law. The romantic literature of the country gives a very different impression, and must be taken largely into account when striking the balance of argument in respect to any people, as it gives the modification induced by the growth of public opinion on the rigour of the law.

Let us now turn to the two greatest nations of antiquity, whose polity and customs have in many ways largely affected our own. We find Grecian women "always in a state of tutelage, perpetually in the power and subject to the direction of their fathers, husbands, or other legally appointed guardians." (*Westminster Review.*) An heiress's son, when he came of age, was empowered to enjoy his mother's estate, allowing her a maintenance. If a woman were cited into court, the form used was —"We cite A B and her guardian," she, alone, being a nonentity. Mr. Grote informs us (vol. vi., page 133) that "the free-citizen women of Athens lived in a strict and almost Oriental recluseness, as well after being married as when single: everything which concerned their lives, their happiness, or their rights, was determined or managed for them by male relatives; and they seem to have been destitute of all mental culture and accomplishments." Women were located at the back of the house, often in the upper part; and it is sufficiently indicative of the excessive strictness of their seclusion, that when the Athenian women stood at the doors of their houses to inquire the fate of their husbands after the defeat at Chæronea, it was considered discreditable to them, *and to the city.* Athenian women, it is true, took part in religious processions, but were then always veiled.

At Sparta the liberty enjoyed was much greater. Only married women wore the veil; maidens went abroad uncovered; and Mr. Grote tells us that "Xenophon and Plutarch represent the Spartan women as worthy and homogeneous companions to the man. The Lykurgean system (as these authors describe it), considering the women as a part of the state, and not as a part of the house, placed them under training hardly less than the men." The reader may here note an exception to the assertion made at the opening of our subject, that in the historical eras women could hardly be said to hold any relation to the *State* at all. Nor was this a political, but rather a physical relation, arising from the great importance assigned to the bodily condition of the Spartan citizens, and consequently to the training of the mothers who bore them. Mr. Grote continues as follows:—

"Female slaves are good enough (Lykurgus thought) to sit at home weaving and spinning, but who can expect a splendid offspring, the appropriate mission and duty of a free Spartan woman towards her country, from mothers brought up in such occupations? Pursuant to these views, the Spartan damsels underwent a bodily training analogous to that of the Spartan youth, being formally exercised and contending with each other in running, wrestling, and boxing, agreeable to the forms of the Grecian agonês. The presence of the Spartan youths, and even of the kings and the body of the citizens, at these exercises, lent animation to the scene. In like manner the young women marched in the religious processions, sung and danced at particular festivals, and witnessed, as spectators, the exercises and contentions of the youths, so that the two sexes were perpetually intermingled with each other in public, in a way foreign to the habits, as well as repugnant to the feelings, of other Grecian states."

The law, however, dealt as hardly by women in Sparta as in other communities. They were disposed of in marriage according to the will of fathers and guardians: if the parent died without determining the fate of his daughter, it became a legal question "to whom, among the various claimants, the best title really belonged." This is analogous to the modification of that early Athenian law by which an heiress and her inheritance belonged to the family, and the consent of the kinsmen was necessary to her marriage; for it was afterwards allowed that her father might dispose of her *by will or otherwise;* but *if* "he died intestate and without male children, his heiress was legally compelled to accept her nearest kinsman, not in the ascending line, as her husband." Nay, it seems that dying husbands could, and did, bequeath their wives to other men.

We must also observe that, in other respects the law and feeling of Athens were "as unjust to women as they are in all barbarous, and we may add, in all civilized countries," adultery being only recognised and punished on the part of the woman, wholly overlooked on that of the man.

We now come to Rome, whose system of laws lies at the foundation of much of our own constitution, and here we are met by the somewhat discouraging fact, that the laws regarding women, rigorous in republican days, gradually expanded into a most remarkable fairness and equality in those very centuries when the empire was approaching a disgraceful decline and fall. It is unpleasant to connect the epoch of some of the worst women the world has ever seen with the very changes we are desirous of effecting in our own country; but since the connexion will probably be used in argument by those who think it advisable to retain our system as it is, it may be well to direct the attention of the student to those other causes which degraded Imperial Rome—the concentration of wealth in metropolitan cities, undue and unjust taxation, the decomposition of an immense unwieldy empire—various causes which are not working in our modern civilization, and which education, and the different industrial position of the female sex, forbid us to imagine can again recur.

We cannot here do better than quote a passage from the "Report of the Personal Laws Committee on the Law relating to the Property of Married Women," lately published by the Law Amendment Society.

"In the earliest period of the Republic, the rights and conditions of married women were entirely subordinated to the absolute power of the head of the family, or *paterfamilias*. The wife passed into the husband's possession under the marriage contract, which pursued the forms of a sale. He had absolute power over her as over a slave, even, as is alleged by some, to life and death. She had no dowry; she could not possess property: and whatever came to her hands immediately became the property of the husband. The injustice of these regulations was, however, felt by the great legislators of the commonwealth."

And the following extract from Fraser, on *Personal and Domestic Relations*, describes the condition of the Roman wife at the best period of their laws:—

"The Roman wife was not held to be sunk in the husband, but after the marriage she remained as capable of independent action as before. Each could possess and enjoy property; and whatever one acquired, the other could have no participation in. The wife's debts could be recovered only from herself, and the husband's were effectual only against his own person and property. But the presumption in any case was in favour of the husband; and unless the wife established by legal evidence that the property was hers, the husband, his heirs, or his creditors, could demand it."

Again,

"The mode in which the independence of a Roman wife, as to property, was maintained, was as follows: Previous to marriage, a portion of the wife's property, called *dos* or dower, was set apart for the expenses of the wedded state. The administration of this settled property was committed to the husband, and, if it were of a perishable nature (res fungibiles), he became absolute owner of it; but, if land, he had no power of alienation, not even with the wife's consent, except under very special circumstances. All her other property, moveable or immoveable, whether acquired before marriage or after, was entirely under her own authority and control, and was called paraphernalia (*bona parapherna*)."

In other respects the laws of Rome changed no less. Women were freed from tutelage, and the change was wrought in great measure by the help of one of the forms of Roman marriage called usus. This provided that the woman passed from the power of her father into that of her husband, by remaining an unbroken year in his house; but if she absented herself for three nights, a *trinoctium*, she remained in her own familia. In this she followed a *general* law of property, of which ownership was acquired by continual possession. The patricians, appreciating the value of the remedy thus afforded in shielding them from some consequences of intermarriage with plebeians, caused a formal recognition to be made by law of "the interruption of possession as a means of preventing the wife from falling into the power of her husband." The annulment of infant betrothal followed, and increased facilities for divorce, giving to women the same defences as to men, till at length the whole code presented the first and last specimen of just legislation on these points that the world has seen.

With the fall of Rome came the destruction of her social system; but wide and sweeping as was the torrent of barbarism, such remnants as the Roman ruins scattered far and wide over Europe, and mixing their stately strength with the Gothic picturesqueness of Arles, Nismes and Avignon, were not the only traces which remained of her influence. We find some apposite remarks in M. Guizot's "Lectures on European Civilization," which we proceed to quote.

"A municipality like Rome might conquer the world, but could not so easily retain and govern it. Thus, when the work appeared completed, when the entire west, and a great portion of the east, became subservient to Rome, this prodigious number of cities, of small states, formed for independence and self-existence, became disunited, and detached themselves in every direction. This was one of the causes which led to the foundation of the empire. It was necessary to change the form of government for one more concentrated, more capable of maintaining union among such discordant elements. The empire attempted to bind together and to unite this widely diffused society. * * * We observe at the fall of the Roman empire the same fact which we recognised at the foundation of Rome, viz., the predominance of the municipal character and government. The Roman world reverted to its pristine condition; it was formed by a confederation of cities, and after its dissolution, the same, or similar cities, remained."

The municipal institution and the idea of an empire, M. Guizot considers to be the elements which Roman civilization has transmitted to that of Europe; "on one side the principle of liberty, on the other that of a general and common civil legislation,—the idea of absolute power,—the principle of order and servitude." To these let us add the example of a code of laws respecting women, which for wisdom and enlightenment have never met with a parallel; and since, in other directions, the revival of Roman law in the middle ages has so powerfully influenced our own, inducing changes in feudal jurisprudence which have gradually penetrated to the roots of our social life, we may hope much from a clear statement and wide dissemination of its principle and practice in regard to the female sex. And however little it may be the custom to think one of as great value as the other, society will assuredly find the condition of half its component elements no less important than any question of town government. That federal system which combines the greatest amount of local action with the most implicit obedience to a central power, as in the example of the United States of America, is acknowledged, (save for the blot of slavery in this particular instance,) to be the ideal dream of politicians, and the legislation which secures to the different members of a human family the most perfect freedom of action, allowing them to move spontaneously around one great central idea of duty, will be found to secure in the end the closest unity and the profoundest peace.

We now come to consider the social ideas of those races which, a few centuries after Christ, poured down upon Rome, till

> "Feeble Cæsars shrieked for aid
> In vain within their seven-hill'd towers."

It was not what we might expect, for we find on all hands that in the earliest times of Teutonic invasion, the women held a position of almost supernatural elevation. The early Germans, we are told by Gibbon,

"Treated their women with esteem and confidence, consulted them on every occasion of importance, and fondly believed that in their breasts resided a sanctity and wisdom more than human. Some of these interpreters of fate, such as Velleda in the Batavian War, governed, in the name of the deity, the fiercest nations of Germany. The rest of the sex, without being adored as goddesses, were respected as the free and equal companions of soldiers; associated, even by the marriage ceremony, to a life of toil, of danger, and of glory. In their great invasions, the camps of the barbarians were filled with a multitude of women, who remained firm and undaunted amidst the sound of arms, the various forms of destruction, and the honourable wounds of their sons and husbands. Fainting armies of Germans have more than once been driven back upon the enemy, by the generous despair of the women, who dreaded death much less than servitude."

Yet this enthusiastic tone of character did not carry its development on into the increasing civilization of the Teutonic race. The elaborate Roman law was destroyed, and it would seem as if the poetical and religious halo described above ceased to afford protection to female interest; for the feudal spirit, moulding the institutions of each Teutonic nation, deprived women of everything like legal independence. Land was held by military tenure, and the law of primogeniture bestowed the family property on the eldest son, leaving the younger ones to carve their own way to fortune, and the daughters dependent on marriage or the convent. Chivalry tempered the despotism of the feudal tenure among the upper classes, but could have little influence over the lower; and till the era of the printing press, when the literature of the ancients was once more disseminated through those countries over which they once held imperial sway, the female sex was left to its unaided influence for any freedom or authority it might possess.

Having traced the legal condition of women in other ages and countries, it is now time to come to our own, and to ask ourselves what is the present condition of those Englishwomen whom it is the custom to regard as the freest of their sex. And here we are met by great contradictions between the law and public opinion; the former encumbered on all hands with the fag ends of feudalism, the latter according year by year a larger share of freedom and of influence in many directions. The body of educated Englishwomen press against the law which encircles them, with a weight and persistency which will eventually change nearly all the legal conditions of domestic life; though its moral ideal, deeply rooted in the inmost heart of a Saxon and Christian nation, shows little symptom, and we thank God for it, of dissolution or decay.

To be brief, the English law of the present day, according full liberty to the unmarried woman past her minority, replaces the wife in the position of a minor, property and person being virtually in the power of the husband. His power over the one is modified by various legal devices by which parents contrive to secure at least the capital of such property as they bestow upon a daughter, to her and her children; and the action of the Courts of Equity can be invoked, by those who possess money and patience, for the protection of bequests and the redress of any flagrant wrong. His power over the other is modified by the Habeas Corpus Act, by various statutes concerning the keeping of the peace, and by a strong and ever increasing force of public opinion, which claims for women, of the lowest as of the highest class, an absolute exemption from personal tyranny, and is even inclined to give the husband a *quid pro quo* for every unmanly blow inflicted upon the person of his wife. The practice of the law diverges ever more widely from the theory of the law, and the whole question is in that confused state of germination in which a different opinion prevails on every side.

The law declares that "a man and wife are one person. The wife loses all her rights as a single woman, and her existence is entirely absorbed in that of her husband. He is civilly responsible for her acts; she lives under his protection and cover, and her condition is called coverture."* Society declares that a man and wife are in many cases essentially two people, each possessing strong individuality, each perhaps practising a different profession or means of getting a livelihood,—perhaps even separated both in the inner and the outer life, and warring at as great a distance as is allowed by the length of their chain. Society, in according education to women, has made them capable not only of managing, but of acquiring property by their own exertions, and has given them the desire to do so. The law declares that "a husband has a freehold estate in his wife's lands during the joint life of himself and his wife—that is to say, he has absolute possession of them as long as they both live." Also, that "money earned by a married woman belongs absolutely to her husband;" and that "what was her personal property before marriage, such as money in hand, money at the bank, jewels, household goods, clothes, &c., becomes absolutely her husband's, and he may assign or dispose of them at his pleasure, whether he and his wife live together or not." Society tries hard to circumvent the law, to contrive strong and cunning settlements, whereby the wife's personal property before marriage may be settled on herself. Society sets up a somewhat complex and crazy machinery to redeem the wife and children out of the power of a bad man, where such happens to be, and is even now making an effort to get that part of the law radically altered which relates to property. And this brings us again to the before-mentioned Report of the Law Amendment Society, which may be considered as the latest and most authentic source of information. It contains in the Appendix various details as to the law in various of the United

* "A Brief Summary in Plain Language of the most Important Laws concerning Women, together with a few Observations thereon." By Barbara Leigh Smith. John Chapman.

States of America, and sums up the result in the following paragraph (page 11):—

"The United States of America, which for the most part adopted the common law of England, some with, some without, the correctives of courts of equity, have, during a long course of years, gradually modified the harshness of the law which denies property to married women. And in the great States of New York and Pennsylvania, as well as in New England, in Texas, California, and the newly settled States, a married woman is allowed, with more or less modification, the same rights over property as if she were single. In the States where the civil law prevailed, the provisions of the Roman code had already secured independence to married women."

The Report also specifies the provisions of the French law, which is much more equal than our own, and concludes with a recommendation that a law of property as to married women should be based on the following principles:—

"1. That the common law rules which make marriage a gift of all the woman's personal property to the husband, to be repealed.

"2. Power in married women to hold separate property by law, as she now may in equity.

"3. A woman marrying without any antenuptial contract, to retain her property, and after acquisitions and earnings, as if she were a *femme sole.*

"4. A married woman, having separate property, to be liable on her separate contracts, whether made before or after marriage.

"5. A husband not to be liable for the antenuptial debts of his wife any further than any property brought to him by his wife under settlement extends.

"6. A married woman to have the power of making a will, and on her death intestate, the principles of the statute of distributions as to her husband's personalty, mutatis mutandis, to apply to the property of the wife.

"7. The rights of succession between husband and wife, whether as to real or personal estate, courtesy or dower, to be framed on principles of equal justice to each party."

It may be long before any such law receives the sanction of the British legislature, but the day must come, as assuredly as that of any of those great reforms which have gradually been incorporated in the statute book.

But the relation of the law to women forms but a small portion of the relation of women to society; and the question is now becoming complicated by new elements which rise into view with every new phase of development afforded by the increased education of the sex. How great a change has been effected since the days when the good and learned Elizabeth Carter was a kind of national prodigy for her translations from the Greek, and concerning whom, a hundred years ago, a report arose in the town of Deal, where she resided, that she was about to be returned as the borough member to Parliament! There are hundreds of women who could now translate Epictetus, and thousands who could write as good poetry as the stately odes and verses perpetrated by Elizabeth Carter, which are perhaps about the level of a University prize poem. A woman of ability possesses, in the present day, advantages in the way of education which in some respects transcend those of men. She has not, it is true, that assistance to a severe mental training which the great schools and universities afford to the other sex, and can seldom, for lack both of teaching and of stimulus, attain to the same accurate technical proficiency in any branch of knowledge as is demanded from an aspirant to one of the learned professions. On the other hand, leisure, freedom from the trammels of one engrossing subject, and keenness of general social sympathy, secure to a really cultivated woman a certain breadth of survey; and where she is not a slave to prejudices of social etiquette, her conversation possesses a universality of interest and a depth of moral insight, which are not often matched, at least in general society, among men possessing even eminent attainments in one or two special directions. This new power may be seen at work on all hands, rousing up discussions on social subjects, striving to force out fresh lines of occupation, creating fermentation in the most orderly homes, and in many cases, where it finds a difficulty in harmonizing with the existing state of things, bringing not peace, but a sword.

No fact is in the present day more noticeable than that the religious systems of England afford no definite or regulated scope for female activity. We have had plenty of exciting convent stories, full of abduction, seduction, and murder; it is now time, in the revolving cycle of opinion, to ask ourselves whether we did well when, at the Reformation, we overthrew all those grand fabrics whose ruins yet stand up in our old towns and amidst our wooded valleys, beautiful even in their decay and desolation. Protestant Germany has its large, orderly hospitals, managed entirely by deaconesses of the Lutheran persuasion,* and Catholicism directs into many fertilizing channels the fund of energy and kindness at its disposal; but in England a hard Puritanism succeeded the downfall of the old faith, alien in its main features to all the distinguishing characteristics of women, which in fact, from the extreme prominence given both to the Jewish and to the Pauline theology, it more than half-despised. We do not ignore the heroic virtues which the Protestant spirit fostered; the brave women of the Commonwealth, nor those who went out

* Vide a little pamphlet by Miss Nightingale on the Deaconesses of Kaiserswerth, an institution founded by Pastor Fliedner.

in the Mayflower to found the present race of New England women, who for energy and intellect are the flower of their kind. But we do believe that much was swept away which it would have been wise to have retained, since religious and benevolent women have from that time struggled, in a disorganized and therefore comparatively ineffectual way, to forward those works of charity and mercy towards the fulfilment of which the Catholic Church affords so much training, help, and encouragement.

In a little volume called "Hospitals and Sisterhoods," the reader will find details as to Catholic Orders of Nuns which it is not very easy to meet with elsewhere; for in Protestant accounts of the Catholic Church, or in histories of the Reformation, is so slight a mention of the organizations for female activity, that it is evident how little their peculiar spirit and work is understood by the writers. The very Encyclopedias which give elaborate accounts of monks and monasteries pass over the word nun with little more than a derivation. Yet in the opening words of Chapter VI. of the little book above-mentioned:—"It would not be possible to give within the compass of these pages the rise, progress, and extent of the religious orders of the Roman Catholic Church. Every talent committed by God to mankind found in them scope for use." On page 92 may be found an account of the Order founded by St. Vincent de Paul; and apropos of this, we read in Ranke's "History of the Popes," vol. ii., page 449, a passage exactly expressive of the marked difference between the practical organization of Catholic, and English Protestant communities. Speaking of the saint in question, the historian observes:—

> "The Order of the Sisters of Mercy also owes its origin to him—an order in which the more delicate sex, at a time of life when all the visions of domestic happiness or worldly splendour float before their eyes, devoted themselves to the service of the sick,—often of the abandoned,—without venturing to give more than a transient expression to those religious feelings which were the source and spring of all their toils."

These efforts for the improvement or the consolation of humanity are more happily become of constant recurrence in every Christian land; the education of the poor, the promotion of learning, the mitigation of human suffering, everywhere command attention. Never will such efforts succeed without a union of varied ability and knowledge with religious enthusiasm. In Protestant countries they are too generally left to the energy of each successive generation, and to a sense of the necessities of the moment. But Catholicism aims at giving an unalterable basis to associations formed for such objects, and an uniform direction to the religious impulse which prompts them, in order that every effort may be consecrated to the immediate service of the

Church, and that successive generations may be trained by a silent but resistless process in the same spirit.

If Miss Nightingale, on her return from the East, should succeed in founding any permanent institution for the training of nurses, she will lay in England the first stone of an edifice which may go far to replace those institutions of whose moral value we deprived ourselves somewhat too hastily, when we confounded in one sweeping indignation both use and abuse.

But it was not only in works of practical benevolence that the Catholic Church developed the energies of the sex : Romanism also afforded large scope for the intellectual powers of women, and that in many ways. Female saints seem to have been in as high favour, and to have been as much quoted on spiritual subjects, as those who bore masculine appellations. The reverence paid to the Virgin may be considered as devoted to her supernatural mission and motherhood ; but the saints were mere women who attained their high spiritual rank by their own endeavours after holiness. We find in a work by the Rev. W. Faber* perpetual reference to the sayings of female saints, to revelations made to them, and to treatises on religious subjects written by them. Father Faber is a convert, and one of the most eloquent of the English Catholic clergy of the day ; and this reference to women is perhaps one of the most marked features of a book whose popularity has been very great.

Apropos of the religious foundations of the middle ages, and the position assigned to women by the Catholic Church, we will here quote an interesting passage from a French work entitled "Histoire Morale des Femmes, par M. Ernest Legouvé." It is well worth a perusal by all who are interested in the subject ; and we are moreover informed in the preface that the ideas upon which the book is based have formed the subject of a public course of lectures delivered by M. Legouvé at the Collège de France:—

"Convents have always been regarded as prisons for women ; and, in truth, no places have heard more sobs and legitimate cries of revolt. Women have, however, only there experienced freedom ; and there only have they been able to show what they were worth. A woman powerful in heart and mind, was stifled in the jail of a German or a feudal marriage ; in the cloister she lived, she acted ; as the superior or head of an order she governed. One who wishes to judge of women, should read the history of the great religious foundations. Worldly goods to administer, souls to guide, regulations to establish, journeys to undertake, law suits to undergo, memoirs to draw up ; all, in fine, which constitutes the mechanism of social, if not political functions, became for them a necessity, and out of this necessity they created a long array of virtues.

* "All for Jesus."

"The Abbey of Fontevraud exhibits, if one may so speak, a whole series of eminent men in the succession of its superior abbesses: the monks found themselves, as is known, face to face with nuns in relations of submission, of deference, even of obedience.

"The abbess bore the title of General of the Order. The abbess alone administered the goods of the community. The abbess only could receive an adept in religion. The abbess decreed civil and ecclesiastical punishments. The abbess chose the confessors for the different houses of the order.

"Did this concentration of administrative powers in the hands of women injure the prosperity of the institution? Not at all. No congregation was richer or more illustrious. Enemies were, however, not wanting. During six hundred years, and under thirty-two abbesses, not one of those privileges but was attacked by masculine pride or violence; not one which was not maintained by the energy of women.

"We cite the institution of Fontevraud, we might cite two hundred others, for this is no question of a few isolated traits, nor of superior women; it is by thousands, and in every age of the modern world, that women have displayed the true qualities of organisers. Let us mention St. Theresa, that poor barefooted Carmelite, as she called herself, full of good desires, but destitute of the means of executing them, and who succeeded alone, and without help, in founding twenty monasteries in Spain. Let us mention Heloise, who in the government of the Paraclete showed, as directress, so noble and delicate a talent. Let us mention the company of daughters of charity, who sometimes went by tens, twenties, and thirties on to the fields of battle to tend the wounded, as in the wars of 1650 and 1658; sometimes set out for foreign countries, in order to strive against some public scourge, as during the great pestilence which depopulated Varsovia in 1652.

"Finally, the history of Port Royal offers to our eyes, among women free to act, all kinds of firm and spirited conduct. So many signs of firmness, of administrative talent, of the sentiment of duty, of the spirit of business, of active charity, of good practical sense, so much merit of all sorts displayed during several centuries by women, *en masse*, in the only social career which was open to them, disposes, it appears to me, of half the question we proposed to ourselves in this chapter. Women should possess a place amidst social functions in the name of social interest itself."

While Miss Nightingale was pursuing in England those studies which resulted in her fitness for the post of public usefulness she has since occupied, and while Miss Sellon was organizing, in spite of opposition, a semi-Protestant institution under the care of the Bishop of Exeter (and doing noble work in the west of England during times of cholera and fever), an attempt was made in America to open the medical profession to women, and it appears to be succeeding, in spite of the strong feeling of opposition which such an idea would naturally at first excite. Yet it is but an extension of the idea of nursing, which brings women to leech – to heal into quite as many painful scenes, and would be, if efficiently carried out, as repugnant to fanciful and effeminate delicacy. The ladies of the middle ages were distinguished for their leech-craft, and nobody accuses them of having stepped beyond their province. The name of Elizabeth Blackwell begins to be well-known amongst those who feel interest in social questions. This lady was born in Bristol, but emigrated with her family to America while yet a child. Being cast upon her own exertions, by family circumstances, she was for a few years a teacher, but the idea of opening out a new path for women having penetrated, and at length engrossed her mind, she made arrangements for commencing the study of medicine in the family of a physician, and finally sought admittance into some regular college. With great difficulty she found one which would receive her, in Geneva College, New York State, and passed some time there amidst much social opposition in the town, but perfect respect and appreciation from professors and students. She took her degree triumphantly, and received the same afternoon innumerable visits from the ladies of Geneva, who had hitherto shunned her! After attaining this platform, she came to Europe, and resided for some months in Paris, during part of which time she was immured in the Hospital of La Maternité, in company with a crowd of French women of the lower class, training as midwives. This she always described as the most irksome association she had to undergo during her studies, infinitely more so than that of the male students of an educated class. She was afterwards permitted to "walk" St. Bartholomew's Hospital in London, and while residing here made many friends among English people. She returned to New York in 1851, and has gradually built up practice and reputation by force of accurate knowledge and a clear commanding character of a very uncommon order. She has published a volume of lectures, which were delivered to a class of ladies in 1852, entitled "The Laws of Life, with special Reference to the Physical Education of Girls," and which may be bought in London from American booksellers. It contains knowledge, thought, and feeling, which secure it an increasing circulation. This lady has a younger sister, Emily, who has pursued nearly the same path of study, and was for some time a pupil to Dr. Simpson, of Edinburgh. It is at present uncertain whether she joins her sister in New York, or attempts to establish a practice in London.

The relation of the female intellect to the arts is another question which is rapidly rising into importance. It is very certain that no women have hitherto shown the least claim to be associated with men on the higher platforms of artistic eminence; unless we except the name of one living artist, Rosa

Bonheur. However much we may make of the name of Angelica Kauffmann, or of those of the few female students scattered among the Italian schools, no honest mind will for a moment assign to them more than a third-rate place. No woman has excelled in sculpture; we can even remember no statue by one, which has attained any degree of celebrity, except that of the Maid of Orleans, by the daughter of Louis Philippe. We trust, however, that Miss Hosmer, the young American sculptress, now studying under Gibson, at Rome, may ere long vindicate her claim to rank as a true artist. No woman has shown a commanding genius in poetry between the dates of Sappho and Elizabeth Barrett Browning,—a sufficiently wide interval, one would think, for the outcoming of any eminent gift of song. The great oratorios, the great operas, even the madrigals and canzonets are all written by men. The music of the heavenly spheres has never, it would seem, been heard aright by women, nor by them translated into the language of earthly sound. So much must be fully granted, for it is true, and is said on all hands, whenever any appeal is made for the thorough professional education of women in the arts. Yet they possess, as a sex, all those qualifications of sensibility and quick perception, all that fineness and glow of temperament which peculiarly distinguish the artist from other men; and when this is balanced and supported by so large a share of practical intellect as women of the present day do undoubtedly exhibit, why is it that they do not excel in the creative arts? Those who hope little of women will reply that nature never intended, and that domestic duties will not allow of the sustained study and hard work necessary for the attainment of proficiency in any art; that women are intended to appreciate painting, music, sculpture and poetry, and to diffuse their influence through society, not to struggle for the bays. Those, on the other hand, who hope much from women, will say that no genius can force its way through such external disadvantages as those which hamper the sex; that the opportunities of study are in all ways restricted; that the great public institutions are closed to them; that even in youth they are hampered by a thousand silken fetters of domestic life, and that any artist really put into the position of a female student would be sorely inclined to throw brush, chisel and harp into the nearest fire that came to hand. But the truth seems to us to be, that any lengthened discussion on this point is useless, for that the question is rapidly narrowing to a practical issue; the facilities for artistic study, though still far behind those of men, are increasing every day; and so many women have entered the artistic career that social disabilities will gradually disappear. The public is also thoroughly willing to appreciate female work, perhaps even to regard it more highly, when it is good, because it is a woman's. Rosa Bonheur's great picture of the Horse Fair was greeted with a generous chorus of admiration, and her claims to rank as equal or superior to Landseer discussed on all hands. Mr. Ruskin holds up the Misses Mutrie as flower painters of the first order; and poor Edgar Poe, in collecting those poems which, headed by the "Raven," have lately attained such general circulation among lovers of literature, gave an American's tribute to our greatest female author in these words:—"To the noblest of her sex—to the Author of "The Drama of Exile"—to Miss Elizabeth Barrett Barrett, of England, I dedicate this volume, with the most enthusiastic admiration, and the most sincere esteem." So that women have no cause to complain of due recognition being withheld from them; they have now but to try the question of their powers fairly in an open field.

We have omitted to mention the dramatic art, in which women at least equal men. At the present day there are no actors who may be classed with Rachel, Ristori, and Charlotte Cushman, while Mrs. Siddons, in the memory of our fathers, claims a pre-eminent place.

We have not yet touched upon literary women and their effect on the age, because it is the most obvious part of our subject, and one on which little can be said that will not have suggested itself to the reader. It is in fiction of a profound and passionate order that they have chiefly excelled; and remarkable it is that when they do take up the pen, it is not to depict the external conventionalities in which women are supposed to abide, or those mild passions which poets feign to reside in the female breast, but to plunge into the deepest mysteries of human life, raking and ploughing into experiences upon which men seldom touch. The names of George Sand and Currer Bell are associated with books which have struck at the very heart of modern society; and Frederika Bremer, though her mind is of a more tenderly sympathetic cast, and softened by that gift of humour which is one of the safety-valves of genius, is fond of uncovering the mouths of social pits into which the stoutest beholder can only look and shudder. Women use their pens as dissecting knives, and lay bare social arteries till the blood spouts up, and common-place readers cry out and say it is all shocking and false; but yet people buy the books, and they are worn to rags and tatters in circulating libraries, and pass through edition after edition in defiance of the reviews. How different from the days of Miss Edgeworth and Jane Austen! from their clear, charming, crystalline pictures of life, dealing so artistically with all on the surface and never penetrating beneath.

In other departments of literature women have not done much of mark, but more and more of the writing of the day falls to their share. In newspapers and periodicals, as editors, as compilers of historical and all manner of other matter, they begin to form a formidable phalanx, and to exercise an increasing influence. And the part they play in the papers, and in particular the wide power exercised by one pen, which still labours on indefatigably in spite of failing health and numbered days,—we allude to that of Harriet Martineau,—suggests the question of what position women will eventually take in relation to the State, for it is evident that while their influence is penetrating in all other directions, it must in time bear influentially on politics. This is the opinion of such men as John Stuart Mill and Herbert Spencer; the latter observes in his "Social Statics,"—

"It is, indeed, said that the exercise of political power by women is repugnant to our sense of propriety—conflicts with our ideas of the feminine character—is altogether condemned by our feelings. Granted, but what then? The same plea has been urged in defence of a thousand absurdities, and if valid in one case is equally so in all others. There was a time in France when men were so enamoured of ignorance, that a lady who pronounced any but the commonest words correctly, was blushed for by her companions; a tolerable proof that people's *feelings* then blamed in a woman that literateness which it is now thought a disgrace for her to be without. It was once held unfeminine for a lady to write a book; and no doubt those who thought it so would have quoted *feelings* in support of their opinion. Yet, with facts like these on every hand, people assume that the enfranchisement of women cannot be right, because it is repugnant to their feelings."

We are, however, trenching on speculations so far ahead of any possible result, that we feel upon dangerous ground, and must pray our readers to remember that there is the widest difference between holding an opinion, based on philosophical grounds however firm and true, and any attempt to force that opinion into facts which are wholly unsuitable for its reception. It is a common sneer to picture "Women in St. Stephen's," but there they never will be. St. Stephen's will have given place to some assembly more orderly and better ventilated, before women take practical part in political life. It is customary to represent theoretical changes as if each were to take place alone. But it is not so, the combinations of society are as infinite and wonderful as the never-repeated patterns of a kaleidoscope; one bit of the system never alters singly, but in new and totally unexpected directions we find adaptations to the ruling idea of the time. Those who care to see the best that can be said on the subject of the enfranchisement of women, will find it in an article contained in the "Westminster Review" for July, 1851, and also in a small tract on the subject published by Chapman.

We have now gone through the various departments of society wherein women do or may find a place, without a word on the cardinal fact of life for both men and women—their domestic relations. In the first place, it is a subject upon which so much has been said or sung, that its usual aspects are pretty well exhausted; in the next place, its profounder theory involves the whole moral and religious condition of both parties, and cannot be compressed into a paper such as this. Our silence, then, is from no sense of their unimportance, but because we accept the household love and the household duties as the most sacred and perfect manifestation of a noble human character, and consider that their due training and fulfilment depend upon deeper causes and principles than we can treat of here.

